# Application of artificial intelligence in cataract management: current and future directions

**DOI:** 10.1186/s40662-021-00273-z

**Published:** 2022-01-07

**Authors:** Laura Gutierrez, Jane Sujuan Lim, Li Lian Foo, Wei Yan Ng, Michelle Yip, Gilbert Yong San Lim, Melissa Hsing Yi Wong, Allan Fong, Mohamad Rosman, Jodhbir Singth Mehta, Haotian Lin, Darren Shu Jeng Ting, Daniel Shu Wei Ting

**Affiliations:** 1 Singapore Eye Research Institute, Singapore, Singapore; 2 Present Address: Singapore National Eye Center, 11 Third Hospital Avenue, Singapore, 168751 Singapore; 3 Present Address: Academic Ophthalmology, School of Medicine, University of Nottingham, Nottingham, UK; 4 Present Address: Zhongshan Ophthalmic Center, Sun Yet Sen University, Guangzhou, China

**Keywords:** Artificial intelligence, Telemedicine, Cataract, Cataract screening, Cataract surgery, IOL calculations, Biometry, Machine learning

## Abstract

The rise of artificial intelligence (AI) has brought breakthroughs in many areas of medicine. In ophthalmology, AI has delivered robust results in the screening and detection of diabetic retinopathy, age-related macular degeneration, glaucoma, and retinopathy of prematurity. Cataract management is another field that can benefit from greater AI application. Cataract  is the leading cause of reversible visual impairment with a rising global clinical burden. Improved diagnosis, monitoring, and surgical management are necessary to address this challenge. In addition, patients in large developing countries often suffer from limited access to tertiary care, a problem further exacerbated by the ongoing COVID-19 pandemic. AI on the other hand, can help transform cataract management by improving automation, efficacy and overcoming geographical barriers. First, AI can be applied as a telediagnostic platform to screen and diagnose patients with cataract using slit-lamp and fundus photographs. This utilizes a deep-learning, convolutional neural network (CNN) to detect and classify referable cataracts appropriately. Second, some of the latest intraocular lens formulas have used AI to enhance prediction accuracy, achieving superior postoperative refractive results compared to traditional formulas. Third, AI can be used to augment cataract surgical skill training by identifying different phases of cataract surgery on video and to optimize operating theater workflows by accurately predicting the duration of surgical procedures. Fourth, some AI CNN models are able to effectively predict the progression of posterior capsule opacification and eventual need for YAG laser capsulotomy. These advances in AI could transform cataract management and enable delivery of efficient ophthalmic services. The key challenges include ethical management of data, ensuring data security and privacy, demonstrating clinically acceptable performance, improving the generalizability of AI models across heterogeneous populations, and improving the trust of end-users.

## Background

In recent years, artificial intelligence (AI) has had a profound and increasing impact on ophthalmology. The field has evolved from the automation of manual tasks, such as processing of ophthalmic images, to machine learning (ML) and deep learning (DL). ML is a subset of AI that allows the automated system to learn from the available data by discovering the best parameters and weights within a general model such as support vector machines (SVM) [1] or random forests [2]. DL on the other hand is a subset of ML that involves deep neural network architectures [3]. Multiple layers of network neurons perform feature extraction, enabling the model to learn high-level features in an incremental manner. This ability has led to a significant breakthrough in performance on various image classification tasks in ophthalmology [4, 5].

In the field of ophthalmology, cataract is the leading cause of treatable blindness, resulting in moderate or severe vision impairment in an estimated 52.6 million people worldwide [6]. This burden is expected to increase substantially as a result of rapidly aging populations. Eyecare services, however, have been unable to expand in tandem, resulting in a shortfall that is becoming increasingly difficult to address [7]. Furthermore, low to middle-income economies have a higher prevalence of cataract-related visual impairment compared to developed countries [6]. These populations are disadvantaged by low socioeconomic status, poor accessibility to healthcare, and other environmental factors [8]. Scalable eyecare services will need to be devised for improving their accessibility to these under-privileged populations.

In this regard, several AI technologies have been developed to aid various aspects of cataract management. They range from screening and diagnosis [9–12] of both adult and pediatric cataracts, optimizing biometry and intraocular lens (IOL) power calculation [13–23], potential application in cataract surgery workflows and training [24–28], to the prediction of posterior capsule opacification (PCO) progression [29, 30]. To date, cataracts are clinically diagnosed by ophthalmologists at the slit-lamp, requiring a face-to-face consultation. Therefore, undiagnosed cataracts remain a huge challenge for many developing countries and rural populations due to a lack of accessibility. An AI-assisted telemedicine platform for the preliminary diagnosis of cataract will overcome barriers to accessibility, and thus alleviate healthcare burden. Timely diagnosis will be important, especially for pediatric cataracts which can lead to irreversible amblyopia.

Considering that the COVID-19 pandemic has severely disrupted ophthalmic healthcare systems, redeployment of the workforce to service frontlines, suspension of face-to-face clinic appointments, and cancellation of elective cataract surgeries have impeded the system’s ability to address eye care needs. For example, in 2019, 1.7 million residents in the US had cataract surgery [31] compared with the COVID-19-induced lockdown in 2020 resulting in a backlog of over 1 million cases, where 97% fewer cataracts surgeries were completed [32]. This was a familiar phenomenon globally, with countries such as India experiencing a 95% drop in cataract surgeries performed after the onset of COVID-19 [33], creating unsustainable backlogs and prolonged surgical waiting times. Due to these unprecedented challenges, healthcare systems globally have begun to explore alternative models such as telemedicine and AI-assisted platforms for cataract management.

Therefore, this article aims to provide a comprehensive review on the application of AI in cataract management. The scope of this article includes screening and diagnosis in the community and outpatient settings, potential intraoperative care and operating theater workflow management, cataract surgical training, aspects of postoperative care, as well as a discussion on future challenges and directions.

## Main text

### Adult cataracts

#### Epidemiology

Based on the Lancet Global Health [34] and WHO statistics [35] in 2020, the global cataract burden is expected to increase, especially with the current COVID-19 pandemic situation given the shortages in healthcare resources and limited access to medical centers.

While the COVID-19 situation may be unprecedented, the challenges regarding availability, accessibility, and affordability of eye care services in developing countries are longstanding. Studies have reported that cataract surgery coverage was at least 40% lower in countries such as Vietnam, Yemen, and Malawi [36]. Moreover, scarce medical resources are primarily located in tertiary centers in urban regions [7], resulting in shortages in specialized ophthalmology services in rural regions [37].

#### Role of AI in cataracts

**Fig. 1:**
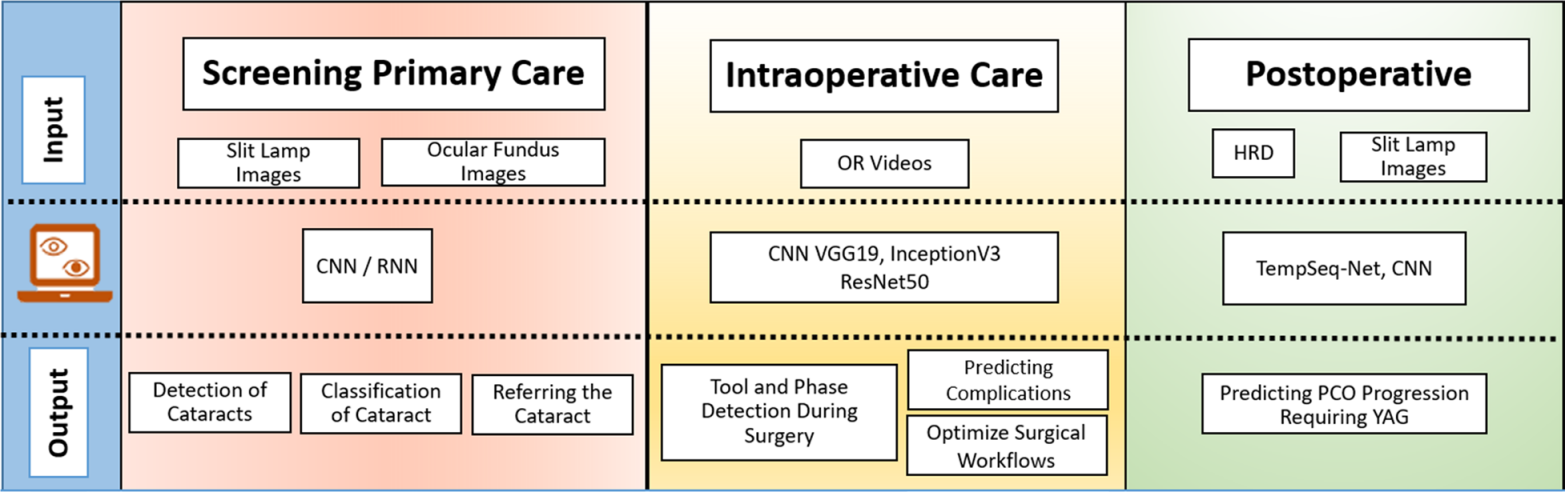
Workflow of Artificial Intelligence in the different stages of cataract treatment. Summary of current and potential AI applications in different stages of cataract management: For screening and diagnosis of cataracts in primary care, slit-lamp images or ocular fundus images are used in algorithms to detect and classify cataracts as well as generate a clinical decision for patient disposition. With regards to intraoperative care, AI models currently use cataract surgery videos to classify the different phases of cataract surgery, which can be applied to predict complications and optimizing surgical workflows. Lastly, for postoperative care, slit-lamp images and health-record data were used to predict PCO progression requiring YAG capsulotomy. CNN, convolutional neural network; RNN, recurrent neural network; OT, operation theater; HRD, health record data; TempSeq-Net, end temporal sequence network; PCO, posterior capsule opacification

AI appears to be promising in this field due to its unique ability to internalize extensive data and analyze large quantities of parameters, even when parameters outnumber observations compared to traditional statistical methods. To date, AI models have been applied to screening and diagnosis of cataracts [9–12], optimization of IOL power calculation for cataract surgery [17–23, 38, 39], classification of phases of cataract surgery from videos [24–27], prediction of surgical procedure timings to optimize operating theater workflows [28], and PCO risk prediction [29, 30] (Table 1, Fig. 1).

**Table 1:** Summary of application of AI in the screening or diagnosis of cataract

Year	Authors	Imaging	Sample size	AI algorithms	AUC	Accuracy (%)	Sensitivity (%)	Specificity (%)
*Adult cataract diagnosis (screening and grading)*								
2020	Li et al. [10]	SLP	1772	ResNet-CNN	–	D = 98.4–99.8	D = 99.4	D = 99.1
2019	Wu et al. [11]	SLP	37,638	ResNet	D = 0.90–1.00 G = 0.86–0.97	D = 84.2–99.5 G = 73.2–94.9	D 60.1–99.5 G = 63.2–92.1	D = 76.4–99.6 G = 63.2–92.1
2019	Xu et al. [12]	FP	8030	AlexNet + VisualDN	–	D + G = 86.2	D + G = 79.8–95.0	D + G = 83.3–88.4
2019	Zhang et al. [75]	FP	1352	SVM + FCNN	–	G = 93.0	D = 99.4 G = 82.4–96.4	–
2017	Xiong et al. [76]	FP	1355	BPNN + MCDA	–	D = 92.8 G = 81.1–83.8	D = 93.1	D = 92.1
2016	Yang et al. [77]	FP	1239	Ensemble learning (SVM + BPNN)	–	D = 92.0–93.2 G = 83.9–84.5	D = 91.4–94.2 G = 62.5–79.5	D = 91.5–92.5 G = 87.9–98.9
2015	Guo et al. [78]	FP	445	MCDA	–	D = 90.9 G = 77.1	–	–
2015	Gao et al. [9]	SLP	5378	CRNN	–	G = 70.7	–	–
2013	Xu et al. [79]	SLP	5378	SVR	–	G = 69.0 (with up to 98.9 for within 1-step error)	–	–
2012	Gao et al. [80]	SLP	434	–	–	D = 62.0	–	–
2011	Cheung et al. [81]	SLP	5547	SVM	D = 0.88–0.90	–	D = 79.7–83.7	D = 79.5–81.9
2010	Acharya et al. [82]	SLP	140	BPNN	–	D = 93.3	D = 98.0	D = 100.0
*Intraocular lens power calculation methods and biometry*								
2021	Ladas et al. [38]	Data	1391	SVR, XGB, ANN	–	PE within 0.5 D = 80.0 (SRK + SVR) 81.0 (SRK + XGB) 67.0 (SRK + ANN) 82.0 (Holladay I + SVR) 82.0 (Holladay I + XGB) 80.0 (Holladay I + ANN) 82.0 (LSF + SVR) 81.0 (LSF + XGB) 81.0 (LSF + ANN) MAE = 0.325 (SRK + SVR) 0.314 (SRK + XGB) 0.439 (SRK + ANN) 0.307 (Holladay I + SVR) 0.309 (Holladay I + XGB) 0.326 (Holladay I + ANN) 0.311 (LSF + SVR) 0.310 (LSF + XGB) 0.319 (LSF + ANN)	–	–
2021	Debellemanière et al. (PEARL-DGS) [39]	Data	6120	SVR, GBT, RM	–	PE within 0.50 D = 87.4 MAE = 0.443 (short); 0.240 (long)	–	–
2020	Carmona et al. (Karmona) [23]	Data	260	SVM-RBF MARS-SOP	–	PE within 0.50 D = 90.4 MAE = 0.240	–	–
2019	Connell et al. (Kane) [83]	Data	846	RM	–	PE within 0.50 D = 77.9 MAE = 0.441 (short); 0.322 (medium); 0.326 (long)	–	–
2019	Wan et al. (Hill-RBF 2.0) [19]	Data	127	RM	–	PE within 0.50 D = 86.6	–	–
2019	Sramka et al. [17]	Data	2194	SVM-RM and MLNN-EM	–	PE within 0.50 D = 82.3–82.7	–	–
2016	Koprowski et al. [48]	Data	173	CNN	–	ECPP 0.16 ± 0.14 Dp	–	–
*Intraoperative tools*								
2020	Lanza et al. [28]	Surgery factors	1229	DA	–	68.4	–	–
2020	Lecuyer et al. [26]	Cataract surgery videos	50	CNN (VGG19, InceptionV3, ResNet50)	–	70.0–84.4	–	–
2019	Yu et al. [24]	Cataract surgery videos	100	SVM, RNN, CNN (SqueezeNet), CNN-RNN	0.71–0.77	91.5–95.9	0.5–97.4	87.7–99.9
*Postoperative assessment of posterior capsule opacification*								
2018	Jiang et al. [29]	SLP	6090	TempSeq-Net	0.97	92.2	81.0	91.4
2012	Mohammadi et al. [30]	SLP	352	ANN	0.71	–	25	97
*Pediatric cataract assessment*								
2020	Lin et al. [51]	SLP	1738	RF ADA	0.86 (RF) 0.85 (ADA)	86.0 (RF) 85.0 (ADA)	80.0 (RF) 77.0 (ADA)	91.0 (RF) 90.0 (ADA)
2019	Lin et al. [52]	SLP	350	CNN	–	D = 87.4 G = 70.8–90.6	D = 89.7 G = 84.2–91.3	D = 86.4 G = 44.4–88.9
2017	Liu et al. [53]	SLP	886	CNN	D = 0.97 G = 0.96–0.99	D = 97.1 G = 89.0–92.7	D = 96.8 G = 90.8–93.9	D = 97.3 G = 82.7–91.1
2017	Long et al. [54]	SLP	1349	CNN	D = 0.92–1.00 G = 0.96–1.00	D = 92.5–98.9 G = 84.6–100	D = 98.8–100 G = 85.7–100	D = 71.4–99.0 G = 90.5–100
2020	Long et al. [55]	HR	594	RF	D = 0.94	D = 89.4–98.1	D = 84.9–98.9	D = 86.9–99.0

*ADA=* adaptive boost modelling; *AI* = artificial intelligence; *ANN* = artificial neural network; *AUC* = area under the curve; *BPNN* = back propagation neural network; *CNN* = convolutional recursive neural network; *CRNN* = convolutional recurrent neural network; *D* = diagnosis; *DA* = discriminant analysis; *DN* = deconvolutional network; *Dp* = diopters; *ECPP* = error of corneal power prediction error; *EM* = expectation–maximization; *EN* = ensemble model; *FCNN* = fully connected neural network; *FP* = fundus photography; *G* = grading; *GBT* = gradient boosted trees; *LSF* = line spread function; *MAE* = mean absolute error; *MARS* = multivariate adaptive regression spline; *MCDA* = multi-class discriminant analysis; *MLNN* = multilayer neural network; *PE* = percentage of eyes; *RBF* = radial basis function; *RF* = random forrest; *RM* = regression model; *RNN* = recurrent neural network; *SLP* = slit-lamp photography; *SOP* = second order polynomials; *SRK* = formula created by John Retzlaff, Kraff and Sanders; *SVM* = support vector machine; *SVR* = support vector regression; *XGB* = extreme gradient boost

#### Screening and diagnosis in cataracts

Gao et al. [9] in 2015 proposed a system to grade cataracts using a combination of convolutional neural network (CNN), recurrent neural network (RNN), and support vector regression (SVR) using slit-lamp images. The algorithm achieved a 70.7% agreement ratio for detecting referable cataracts. On the other hand, Li et al. [10] proposed to identify and annotate cataracts in addition to other anterior segment pathologies with a proprietary model (Visionome) using slit-lamp images. The performance of Visionome was comparable to ophthalmologists while achieving superior performance compared to an ophthalmologist with 1 year of clinical experience (accuracy 79.47–99.22%).

Wu et al. [11] constructed a ResNet DL algorithm with a 3-step sequence in diagnosing and referring cataracts. First, the algorithm recognized the different capture modes of slit-lamp photography and classified the pupil as mydriatic or non-mydriatic (area under receiver operating characteristic curve [AUC] > 0.99). It then differentiated between a cataractous lens, IOL, or a normal crystalline lens (AUC > 0.99). This was followed by cataract severity grading and determination of disposition (referable versus non-referable), achieving an AUC > 0.91. Wu and colleagues [11] also created a telemedicine platform where patients can submit smartphone photos of their eyes. The algorithm determines if they would need to be referred to a healthcare facility. In another study, Xu et al. [12] designed a CNN-based ensemble algorithm (AlexNet and VisualDN) using fundus photos as input to detect and grade cataracts, achieving an accuracy of 86.2%. This concept of using fundal photo clarity as an alternative to anterior segment imaging was mainly motivated by the accessibility of fundus cameras. Through this, automated cataract grading can be scaled without the need for an experienced operator.

#### IOL power calculation and biometry

Precise postsurgical refractive outcomes have been a long-standing endeavor of cataract surgeons. Yet despite significant advancement in IOL formulas, patients with a history of refractive surgery, extreme or atypical biometry still present a challenging conundrum. In the event of a refractive surprise, further interventions such as IOL exchange may be required, which can be distressing for both the surgeon and the patient [40]. Furthermore, IOL exchange can be challenging and exposes the patient to additional surgical risks and complications. Thus, newer IOL formulas are constantly being developed to improve accuracy of predicted refractive outcomes.

Today, with DL, IOL formulas are taking advantage of AI to enhance prediction outcomes. The SVM regression model and multilayer neural network ensemble model (MLNN-EM) evaluated by Sramka et al. [17] showed that both ML algorithms achieved better prediction accuracy than conventional clinical methods. Ladas et al. [38] looked at using AI to improve existing IOL formulae (SRK, Holladay I, and Ladas Super formula), where supervised learning algorithms (SVR, extreme gradient boost [XGB], and ANN) were combined with the above existing formulae to refine the predicted refractive outcome. The hybrid-AI formulas significantly improved the mean absolute error and percentage of eyes within 0.5 D for each of the IOL formulae tested (e.g., SRK with XGB increased the percentage of eyes within 0.5 D to 81% compared to SRK alone [61%]).

Additionally, new formulas have been developed that are either AI-based or utilize AI-incorporated elements. These AI formulas have a promising future, as many have shown high prediction accuracy comparable to established formulas (Table 1). The Kane formula is based on theoretical optics and incorporates both regression and AI components to refine predictions [18]. It has emerged as one of the best performing formulas in comparison studies, exceeding Barrett Universal II, Haigis, Olsen, and other third-generation formulas [13–15, 41]. It has consistently been a top 3 performer even amongst newer generation formulas, and these results were also applicable to both extremes of axial lengths [13–15].

The Hill-radial basis function (RBF) is an ANN IOL calculator that uses regression analysis with a big dataset of refractive outcomes. It employs pattern recognition and data interpolation to predict refractive outcomes [19]. Hill-RBF 2.0 performed better than traditional formulas but was less accurate than the modern formulas (Kane, Evo 2.0, Barrett Universal II, VRF-G) [13]. Hill-RBF 3.0 was recently released with its database significantly expanded to include extremes of axial lengths, while increasing the number of parameters used for IOL power selection. It subsequently showed excellent prediction accuracies similar to new generation formulas (Barrett Universal II, Evo 2.0, and Kane) [20].

The PEARL-DGS formula uses ML modeling and output linearization to predict effective lens position and adjust for extreme biometric values. In a comparison study across 13 formulas [13], it achieved an overall good result, although it was ranked behind the latest generation formulas (Kane, Evo 2.0, VRF-G, and Barrett Universal II). However, when given precise IOL geometric information by the IOL manufacturer, the PEARL-DGS formula outperformed the Evo 2.0, Barrett Universal II, and Olsen formulas [39].

The Ladas Super formula [42] is an IOL formula aggregator that incorporates SRK/T, Hoffer Q, Holladay I, Holladay I with Koch adjustment, and Haigis formulas to create a “super formula”. The super formula comprises ideal portions of the existing formulas derived from a 3-D “super surface”, which was a graphical representation of the most accurate output portions of each IOL formula. Initial results showed that this super formula failed to yield more accurate predictions than the Barrett Universal II or Holladay I [21]. A Ladas Super formula 2.0 is currently being developed that incorporates AI using the big-data approach and will be applicable in the near future [22].

Another new data-driven IOL power calculation method is the designated Karmona [23]. It uses different ML models (e.g., K-Nearest Neighbor, ANN, SVM and random forest) with specific preoperative parameters to predict IOL power. Although it used a small dataset of 260 eyes, it had promising results with superior prediction accuracy compared to the Barrett Universal II and other third-generation formulas.

Apart from those with extremes of biometry, patients who have undergone refractive surgery are at increased risk of refractive surprise as well [43]. Postrefractive surgery alters the normal keratometric index, and thus leads to inaccurate estimation of the effective lens position [44, 45]. As such, much development has been made to precisely measure corneal refractive power. Recently, a new parameter called total keratometry (TK), which is measured using swept-source optical coherence tomography technology on the IOLMaster 700 version 1.70 (Carl Zeiss Meditect AG, Jena, Germany) [46], has been shown to significantly increase prediction accuracy when combined with current IOL formulas [47]. However, further improvement will be beneficial for this increasing segment of patients with high visual demands. AI can therefore be used to augment the evaluation of corneal power. In a study by Koprowski et al. [48], they utilized ANN with error backpropagation on 172 patients who had undergone myopic refractive surgery to predict preoperative corneal power from Pentacam parameters, with a reported low error of 0.16 ± 0.14 D. Through this, AI can be a promising aid in determining accurate corneal power and prediction outcomes for postrefractive surgery patients.

### Pediatric cataracts

#### Epidemiology

Pediatric cataracts are responsible for 5–20% of pediatric blindness worldwide [49]. It is the second cause of visual impairment in those under 18 years old in low-income countries and the third cause of visual impairment in children in high-income countries [50].

#### Screening and diagnosis

Visually significant pediatric cataracts should be treated promptly to avoid irreversible amblyopia. Pediatric patients with cataracts face similar accessibility issues as adults due to the need for detailed slit-lamp examination. As such, diagnosis is often delayed for patients who do not have easy access to specialist centers. The time-sensitive nature of the treatment of pediatric cataracts further contributes to the importance of timely detection and prevention. In this respect, recent developments in AI have shown promising results and can help address these issues.

Lin et al. [51] developed a novel AI screening model to identify infants with a high risk of developing congenital cataracts (CC). A set of 11 non-imaging risk factors (e.g., family history and preterm delivery) were analyzed using random forest and adaptive boosting algorithms to identify predictive factors. The CC identification model showed good discriminatory ability to identify cases with CC (AUC 0.94–0.96), demonstrating the potential to serve as a complementary screening tool, especially in under-developed and remote areas.

Concerning diagnosis, a highly accurate ML platform named “CC cruiser” was developed by Zhongshan Ophthalmic Center [52–54], capable of diagnosing, grading, and initiating therapeutic decisions to manage pediatric cataracts. “CC cruiser” uses a CNN algorithm to grade and diagnose cataracts on slit-lamp images [53] and was previously validated using specific datasets with high diagnostic accuracy of 98% [54]. In a multicenter randomized controlled trial [52], “CC cruiser” achieved a reasonable accuracy for cataract diagnosis (87.4%) and treatment determination (70.8%) although it was lower than experienced pediatric ophthalmologists (99.1% and 96.7%, respectively). However, the time to diagnosis from “CC cruiser” was about three times shorter compared with pediatric ophthalmologists, and it achieved a high level of patient satisfaction from reduced waiting time. Hence, AI technology can provide an alternative model of care that reduces workload and provide time- and cost-effective management.

#### Follow-up for pediatric cataracts

It has been recognized that postoperative care of CC requires long-term follow-up to review complications such as aphakic glaucoma and visual axis opacification. This places a burden on traveling with incremental costs incurred by the patients. Long et al. [55] used Bayesian and DL algorithms to create “CC-guardian”, an AI platform that comprises a prediction module to first identify patients at high risk of postoperative complications then schedules a follow-up visit at a primary care center based on the risk stratification, and finally, utilizes a telehealth computing module to make a clinical decision regarding treatment options (referral to a specialized care center versus continual primary care follow-up). The model achieved a high level of specificity and sensitivity and marks a breakthrough in the way ophthalmic care can be delivered. If AI prediction models and telehealth computing can eventually achieve clinical implementation, it can free up scarce medical resources and reduce travel burden and healthcare costs.

### Intraoperative

Similarly, AI can augment cataract surgery training, intraoperative decision-making, and provide postsurgical analysis to enhance surgical approaches.

In a recent study, an AI algorithm was able to appropriately classify the different phases of cataract operations [25]. Phase classification evolved from automated surgical tool detection [27] to automated phase detection on cataract surgery videos [26]. Yu et al. [24] found that the best accuracy for the automated identification of phases in cataract surgery was to model instrument labels (alone or with video images) rather than with video images alone using either CNN, RNN, or SVM algorithms. The detection of different phases of cataract surgery (e.g., capsulorrhexis, cortical removal, lens insertion) can potentially translate into phase-specific assessments of surgical technical skills and enable procedure-tracking during surgery. This will allow real-time feedback and augment intraoperative decision-making [56].

AI may be applied to predict the risk of intraoperative complications and optimize surgical workflows. Lanza et al. [28] customized an AI model analyzing 1229 surgeries with 73 missteps to detect risk factors for intraoperative complications and predict overall surgical duration. Diaglinear discriminant analysis was used to identify the main risk factors associated with intraoperative complications in a particular hospital unit. These initial findings support upcoming development of customized AI models to predict surgical complications. Regarding the prediction of surgical timings, neural networks were used with backpropagations. There was no statistically significant difference between the predicted and actual mean surgical time in the study population (with an error of < 6 min). In the future, these models can be refined to facilitate scheduling or resource distribution in order to maximize operating theater efficiency.

AI and virtual reality can be used in tandem to develop intelligent teaching systems for cataract surgical training. The viability of such an approach for microsurgical training has already been assessed in the neurosurgical discipline. Mirchi et al. [57] created a virtual training platform (using SVM) where trainees were assessed on a virtual neurosurgical procedure against experienced surgeons. During the training, the trainees were guided using targeted verbal and video-based feedback. Similarly, commercially available ophthalmological simulation-based training machines, such as Eyesi (Haag-Streit, Köniz, Switzerland) [58], can harness AI in a similar fashion to provide a comprehensive training experience, allowing trainees to gain proficiency before actual patient exposure.

### Postoperative

Among cataract surgery complications, PCO is the most common and visually significant [59, 60]. Mohammadi et al. [30] first proposed a prototype ANN by selecting ten input variables to construct a decision tree that predicted PCO requiring capsulotomy with reasonable accuracy (87%) and AUC (0.71). Jiang et al. [29] later demonstrated a more effective prediction of PCO progression requiring YAG laser capsulotomy (AUC 0.97, accuracy 92.2%) by using an end-to-end temporal sequence network (TempSeq-Net) that employs CNN and long short term memory (LSTM), based on 6090 slit-lamp images. This can potentially guide treatment planning by identifying at-risk individuals, thereby avoiding unnecessary visual compromise.

### Challenges and future directions

AI has the potential to be a useful adjunctive tool for cataract management. However, several challenges and concerns will need to be addressed for successful translation. The datasets involved should ideally be heterogeneous to achieve a robust degree of generalizability. The significance of this was illustrated by Ting et al. [5] in a study for diabetic retinopathy detection. The model was trained using data from a multiethnic cohort, achieving a clinical performance comparable to human graders (AUC = 0.93) [5]. However, this often requires sharing of sensitive medical data which contravenes regulations, such as the General Data Protection Regulation (GDPR) in Europe and the Health Insurance Portability and Accountability Act (HIPAA) in the US. Additionally, data security is a common concern during the development of AI models. Aggregation of large amounts of sensitive medical data can constitute a single source of failure. Currently, medical data is a frequent target for hackers [61], particularly in Europe and the US [62], with healthcare continuing to incur the highest average breach costs of US$7.1 million [63]. Adversarial attacks may also exploit these models, either by injecting compromised data during training (data poisoning) [64] or altering input images, to induce large-scale misclassification of the AI model [65]. Furthermore, trust of end-users (physicians and patients) in these models is critical to achieve successful clinical translation. This requires improvements to the explainability of the AI models, including clear demonstration of the decision-making process.

Various approaches have therefore been proposed to address these issues. Firstly, federated learning is increasingly being employed to allow cross-institution or cross-border AI training without data sharing. This is a privacy-preserving technique that exposes the model to heterogenous non-independent and identically distributed data [66]. An extension known as swarm learning [67] can allow the AI model parameters to be further decentralized, thereby aiding in the development of a generalizable model. Secondly, these datasets can be further expanded using generative adversarial networks, especially for rarer diseases [68] such as CC. To increase the explainability of these AI models, methods include saliency heatmaps highlighting regions of interest together with estimates of the uncertainty involved [69] as well as predefined models or feature extractions [70, 71].

In addition, apart from biometry and certain cataract and PCO screening models [11, 29, 51], many AI models have not achieved a level of accuracy that is clinically acceptable, and further development is needed. Moreover, most of the intraoperative AI reports discussed have focused on emerging technologies without clear clinical application, and much of its use is still hypothetical. However, it is still possible that upcoming developments can deliver practical applications that are not immediately apparent.

Furthermore, we recognize that there still exists significant challenges in the implementation of AI, particularly in developing countries due to poor infrastructure, lack of data availability, funding, and technical expertise [72, 73]. There is also a need for proper screening programs to be first implemented to ensure a wide catchment for the population, without which, AI triaging applications will see limited uptake. Therefore, obtaining governmental support in setting new regulations and policies, and applying low-cost approaches to acquiring and analyzing data sources will be key to establishing AI initiatives in these countries.

Finally, the new technology requires compliance to open standards to ensure transparency and completeness. It can be achieved through strict adherence to recently introduced protocols such as the CONSORT-AI (Consolidated Standards of Reporting Trials for AI) and SPIRIT-AI (Standard Protocol Items: Recommendations for Interventional Trials—Artificial Intelligence) [74]. Their objective is to give AI interventions a common ground to evaluate effectiveness by regulatory authorities and the broader medical community.

## Conclusions

The advent of AI can potentially transform the management of cataract in terms of assessment and monitoring, IOL calculation, intraoperative feedback, and postoperative care. AI has been utilized clinically for IOL calculations, achieving superior results compared to conventional methods. Successful clinical translation can deliver long-term benefits especially for low-income populations, including healthcare efficiency, accessibility, scalability as well as reduced expenditure. To achieve this, several challenges will need to be addressed, including ethical management of data, guaranteeing security and privacy, demonstrating clinically acceptable performance, improving generalizability across heterogeneous populations, and increasing user-acceptance.

## Data Availability

Not applicable.
