# Correction to: Application of artificial intelligence in cataract management: current and future directions

**DOI:** 10.1186/s40662-022-00283-5

**Published:** 2022-03-11

**Authors:** Laura Gutierrez, Jane Sujuan Lim, Li Lian Foo, Wei Yan Ng, Michelle Yip, Gilbert Yong San Lim, Melissa Hsing Yi Wong, Allan Fong, Mohamad Rosman, Jodhbir Singth Mehta, Haotian Lin, Darren Shu Jeng Ting, Daniel Shu Wei Ting

**Affiliations:** 1 Singapore Eye Research Institute, Singapore, Singapore; 2 Singapore National Eye Center, 11 Third Hospital Avenue, Singapore, 168751 Singapore; 3 Academic Ophthalmology, School of Medicine, University of Nottingham, Nottingham, UK; 4 Zhongshan Ophthalmic Center, Sun Yet Sen University, Guangzhou, China

## Correction to: Eye and Vision (2022) 9:3 https://doi.org/10.1186/s40662-021-00273-z

After publication of the original article [1] it was brought to our attention that author Wei Yan Ng was incorrectly included as Wei Yan Yan Ng. The correct name is included in the author list of this erratum and has been updated in the original article.
